# Goal-directed therapy reduces fluid balance while maintaining hemodynamic stability in intraoperative management of pancreaticoduodenectomy: a retrospective comparative study

**DOI:** 10.1186/s40981-017-0144-z

**Published:** 2018-01-08

**Authors:** Satoshi Ishihara, Takeshi Yokoyama, Katsuyuki Katayama

**Affiliations:** Department of Anesthesia, Teine Keijinkai Hospital, Sapporo, Japan

**Keywords:** Intraoperative hemodynamic management, Goal-directed therapy, Pancreaticoduodenectomy

## Abstract

**Background:**

Goal-directed therapy (GDT) is beneficial for surgical patients, especially for those undergoing high-risk surgery. However, little has been reported on the hemodynamic effects of GDT in extensive surgery. We conducted a study to determine the impact of GDT on intraoperative management of extensive surgery.

**Findings:**

We retrospectively collected data from 90 patients who underwent pancreaticoduodenectomy: 44 who received intraoperative GDT (GDT group) and 46 who received conventional hemodynamic management (control group). Intraoperative use of fluids and catecholamines and physiologic variables, including mean arterial pressure, heart rate, and urine output, were compared. We also examined the correlation between the amount of fluid administered and urine output. The amount of fluid administered was comparable, and urine output was significantly larger in the GDT group than in the control group. Fluid balance was significantly smaller in the GDT group (49.7 versus 61.7 mL/kg; 95% confidence interval, − 19.5 to − 4.6 mL/kg; *P* = 0.0019). There was a trend toward higher mean arterial pressure in the GDT group despite lower fluid balance. We found a rank correlation between the amount of fluid administered and urine output in the GDT group (rank correlation coefficient, 0.68; *P* < 0.001), but there was no such correlation in the control group.

**Conclusions:**

GDT increased urine output and decreased fluid balance while maintaining hemodynamic stability. The amount of fluid administered and urine output were correlated in the GDT group.

## Findings

### Introduction

Intraoperative hemodynamic management is related to postoperative outcomes [1]. Goal-directed therapy (GDT) is a framework of hemodynamic management in which fluids and catecholamines are titrated based on indices derived from advanced hemodynamic monitoring. Existing data show that GDT reduces postoperative complications and shortens the length of hospital stay and that it is more effective in high-risk surgery [1, 2]. The supporting evidence is strong enough for several academic institutions to recommend GDT for major surgery [3–5].

Pancreaticoduodenectomy (PD) is a major abdominal surgical procedure associated with long duration of surgery, a large amount of administered fluid, and a high rate of postoperative complications [6]. Although the guideline for enhanced recovery after surgery (ERAS) recommends GDT for intraoperative management of PD [3], this is based mainly on extrapolation from studies of colorectal surgery. In fact, cardiac output monitoring and GDT are not widely used in usual practice [7], and thus, little has been reported on the effect of GDT in such extensive surgery.

We conducted this study to analyze changes in intraoperative hemodynamic management and physiologic variables after implementation of GDT in patients undergoing PD.

## Materials and methods

We investigated the impact of GDT on intraoperative hemodynamic management and physiologic variables compared with historical controls in patients undergoing PD at Teine Keijinkai Hospital, a 600-bed tertiary care hospital in Sapporo, Japan. The study was reviewed and approved on December 24, 2015, by the Teine Keijinkai Hospital institutional review board. Due to the retrospective design of the study, the need for informed consent was waived by the board.

### Study subjects

Perioperative data from consecutive patients who underwent PD electively between July 2013 and June 2015 were analyzed. Patients who underwent an operation for other organs concurrently with PD and patients with renal failure requiring hemodialysis were excluded from the study.

Patients who underwent PD in the 1-year period between July 2014 and June 2015 were designated the GDT group, which represents the first patients who received GDT in this hospital. Patients who underwent PD in the preceding 1-year period (between July 2013 and June 2014) were designated the control group.

### Intraoperative management

All patients underwent the operation under general anesthesia with tracheal intubation combined with epidural anesthesia. General anesthesia was maintained with propofol or an inhalational agent (sevoflurane or desflurane), with or without remifentanil. The epidural catheter was placed at Th 8/9, Th 9/10, or Th 10/11 interspace, and 0.2–0.375% ropivacaine was infused during surgery with varying doses at the discretion of the caregiving anesthesiologist. A radial artery cannula was placed after tracheal intubation.

**Fig. 1:**
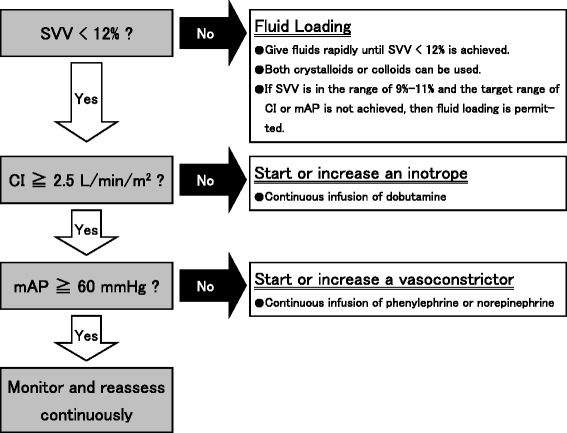
The protocol of goal-directed therapy

Patients in the GDT group received hemodynamic management based on a predefined GDT protocol. The radial artery cannula was connected to a FloTrac sensor and a FloTrac monitor EV-1000 version 1.5 (Edwards Lifesciences, Irvine, CA, USA). First, fluid was given until the goal of stroke volume variation (SVV) < 12% was achieved. At this point, continuous infusion of dobutamine was started or increased if the cardiac index was < 2.5 L/min/m^2^. If SVV and cardiac index were within the target ranges but mean arterial pressure (MAP) was < 60 mmHg, continuous infusion of norepinephrine or phenylephrine was started or increased. The patients were monitored and reassessed continuously and catecholamines were titrated, if used, to maintain values within these predefined target ranges during surgery (Fig. 1). The patients were mechanically ventilated with tidal volumes of 7 to 10 mL/kg and positive end-expiratory pressure ≤ 6 cmH_2_O. Maintenance fluid was restricted to < 5 mL/kg/h.

Patients in the control group received hemodynamic management entirely at the discretion of the caregiving anesthesiologist.

### Data collection and statistical analysis

We assessed intraoperative use of fluids, catecholamines, and blood products; intraoperative MAP and heart rate (HR); estimated blood loss; and urine output. Fluid balance was defined as the total volume of administered fluid and blood products minus estimated blood loss during surgery minus urine output. MAP and HR were recorded just before induction of anesthesia as the baseline and every 30 min after the surgical incision. Analysis of intraoperative MAP and HR was restricted to the period of 270 min after the surgical incision during which all patients underwent the operation.

We expressed dichotomous or categorical variables as numbers (percentage) and continuous variables as means ± standard deviation or medians (interquartile range), as appropriate. The amount of fluid administered, urine output, estimated blood loss, and fluid balance were expressed as values divided by body weight, because body weight was 8% lower in the GDT group.

For dichotomous or categorical variables, we compared frequencies using Fisher’s exact test. For continuous variables, we compared differences using Welch’s *t* test or the Mann–Whitney *U* test when the data were skewed (i.e., urine output). For MAP and HR, we compared differences using repeated measures analysis of variance (ANOVA) with degrees of freedom correction using the Huynh–Feldt or Greenhouse–Geisser estimates of sphericity, as appropriate. We calculated 95% confidence intervals (CIs) of the mean differences for the main results. As the major contributors to fluid balance, we analyzed the correlation between the amount of fluid administered and urine output, using the scatter plot and Spearman’s rank correlation coefficient method. This statistical method was used because of the skewed distribution of urine output.

All statistical tests were two-sided, and we considered *P* values < 0.05 to indicate statistical significance. All statistical analyses were performed with EZR version 1.36 (Saitama Medical Center, Jichi Medical University, Saitama, Japan), which is a graphical user interface for R version 3.4.1 (The R Foundation for Statistical Computing, Vienna, Austria) [8].

## Results

**Fig. 2:**
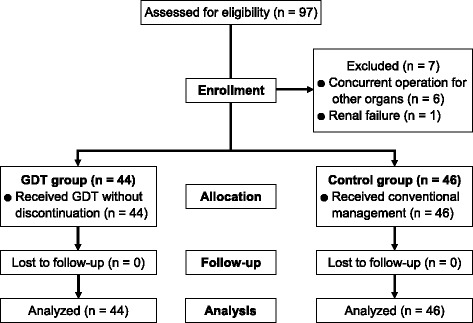
Flow diagram depicting patient enrollment and analysis

A total of 97 patients underwent PD electively in the study period; seven patients were excluded from the analysis based on the exclusion criteria. Consequently, the final analysis included 44 patients in the GDT group and 46 patients in the control group (Fig. 2).

### Demographic characteristics

Most patients were elderly, with a mean age of 67 years. The average duration of surgery was about 7 h. All patients received general anesthesia combined with epidural anesthesia. Body weight was 5.1 kg (8.6%) lower and body mass index was 1.8 points (7.8%) lower in the GDT group. These differences are presumably due to differences in the use of preoperative chemotherapy. Other demographic characteristics did not differ significantly between the groups (Table 1).

**Table 1:** Patient demographic characteristics, anesthetic technique, and surgical parameters

	Control group *n* = 46	GDT group *n* = 44	*P* value
Age (years)	66 ± 12	68 ± 9	0.28
Sex (male)	29 (63%)	26 (59%)	0.83
Height (cm)	160.2 ± 8.7	159.3 ± 9.6	0.65
Weight (kg)	59.1 ± 10.2	54.0 ± 10.5	0.022
BMI (kg/m^2^)	23.0 ± 3.4	21.2 ± 2.9	0.007
Patients receiving preoperative chemotherapy	16 (35%)	21 (48%)	0.28
Preoperative renal function			
Serum creatinine (μmol/L)	61 ± 18	56 ± 17	0.13
eGFR (mL/min/1.73m^2^)^*^	85 ± 25	92 ± 25	0.20
ASA PS			
1	13 (28%)	7 (16%)	0.26
2	27 (59%)	27 (61%)	
3	6 (14%)	10 (23%)	
4	0	0	
Anesthetic agent			
Propofol	29 (63%)	23 (52%)	0.39
Inhalational	17 (37%)	21 (48%)	
Duration of surgery (min)	432 ± 78	434 ± 80	0.92
Estimated blood loss (mL/kg)	9.8 ± 7.5	9.3 ± 5.0	0.71

Data are presented as mean ± standard deviation or number (percentage)

*ASA PS* American Society of Anesthesiologists Physical Status, *BMI* body mass index, *eGFR* estimated glomerular filtration rate

^*^eGFR was calculated using the modification of diet in renal disease (MDRD) equation

### Use of fluids, blood products, and catecholamines

The amount of fluid administered during surgery in the GDT group was 76.0 ± 23.1 mL/kg, which was 5.4 mL/kg (6.6%) less than that in the control group. The difference was not statistically significant (95% CI, − 15.1 to 4.3 mL/kg; *P* = 0.27). The number of patients who received blood product transfusions did not differ significantly between the groups. In the GDT group, significantly more patients received intraoperative continuous infusion of inotropes (34 versus 13% in the control group; *P* = 0.025) and vasoconstrictors (41 versus 7% in the control group; *P* < 0.001) than in the control group (Table 2).

**Table 2:** Summary of hemodynamic management, urine output, and fluid balance

	Control group *n* = 46	GDT group *n* = 44	*P* value (95% CI of mean difference)
The amount of fluid administered (mL/kg)	81.4 ± 23.3	76.0 ± 23.1	0.27 (− 15.1 to 4.3)
Patients receiving transfusion	2 (4.3%)	3 (6.8%)	0.67
Patients receiving infusion of inotropes	6 (13.0%)	15 (34.1%)	0.025
Patients receiving infusion of vasoconstrictors	3 (6.5%)	18 (40.9%)	< 0.001
Urine output (mL/kg)	9.1 (5.4 - 11.5)	11.4 (7.3 - 20.5)	0.025
Fluid balance (mL/kg)	61.7 ± 19.2	49.7 ± 16.3	0.0019 (− 19.5 to − 4.6)

Data are presented as mean ± standard deviation or number (percentage). Use of dopamine in control group was counted as use of inotropes

*CI* confidence interval

### Urine output and fluid balance

The amount of urine output during surgery in the GDT group was 11.4 mL/kg, which was 2.3 mL/kg (25%) greater than that in the control group (*P* = 0.025, Mann–Whitney *U* test). Fluid balance during surgery in the GDT group was 49.7 ± 16.3 mL/kg, which was 12.0 mL/kg (19.4%) less than that in the control group (95% CI, − 19.5 to − 4.6 mL/kg; *P* = 0.0019) (Table 2).

### Intraoperative MAP and HR

**Fig. 3:**
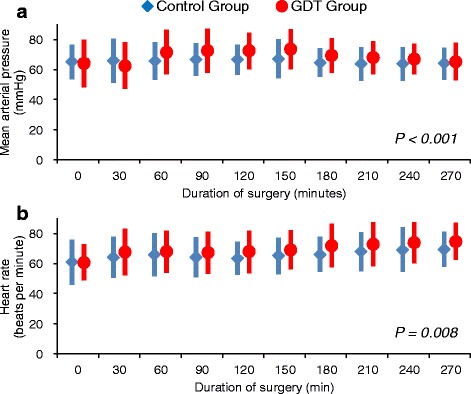
Mean arterial pressure and heart rate during surgery. Filled diamonds and circles indicate means, and error bars indicate standard deviations. **a** Mean arterial pressure. **b** Heart rate. *P* values were calculated using repeated measures ANOVA

MAP and HR, immediately before induction of anesthesia, did not differ significantly between the groups (MAP, 95 ± 14 mmHg in the GDT group versus 98 ± 12 mmHg in the control group; HR, 70 ± 13 beats per minute in the GDT group versus 73 ± 17 beats per minute in the control group). We found a trend toward higher intraoperative MAP in the GDT group than in the control group and a significant effect of GDT (*P* < 0.001, repeated measures ANOVA, *ε* = 0.92). Also, we found a trend toward higher intraoperative HR in the GDT group and a significant effect of GDT (*P* = 0.008, repeated measures ANOVA, *ε* = 0.52). These differences are illustrated in Fig. 3.

### Relationship between amount of fluid administered and urine output

**Fig. 4:**
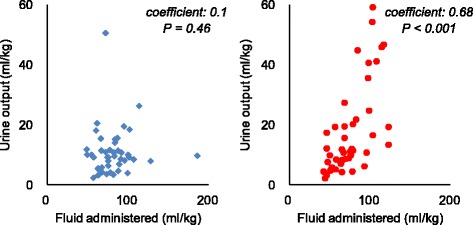
Relationship between the amount of fluid administered and urine output. **a** Control group. **b** GDT group. Coefficient and *P* values were calculated using Spearman’s rank correlation test

Scatter plots of the amount of fluid administered and urine output during surgery in each group are shown in Fig. 4a, b. We found a rank correlation between the amount of fluid administered and urine output in the GDT group (rank correlation coefficient, 0.68; *P* < 0.001). There was no such correlation in the control group (rank correlation coefficient, 0.11; *P* = 0.46).

## Discussion

The present study had two major findings. First, GDT significantly increased urine output and reduced fluid balance while maintaining hemodynamic stability. Second, urine output was correlated with the amount of fluid administered in the GDT group.

The first important finding is that GDT significantly increased urine output and reduced fluid balance while maintaining MAP and HR in patients undergoing PD. Reduction of fluid balance can be reasonably attributed to a shift in fluid management from the conventional method, in which large volumes of fluid are administered in a relatively fixed manner, to GDT, in which the administration of fluid is guided by SVV, a reliable predictor of fluid responsiveness [9].

Some, but not all, previous studies have found similar effects. Reductions in the amount of fluid administered and fluid balance were reported in a quality improvement study that investigated the impact of GDT implementation in a real practice setting [10]. In contrast, no significant differences in the amount of fluid administered or the use of catecholamines were reported in the OPTIMISE trial, which is the largest clinical trial investigating the effect of GDT on postoperative outcomes [11]. Interestingly, the former study found a shortened length of hospital stay in the GDT group and concluded that GDT improves postoperative outcomes, but the latter study found no significant effect of GDT in a composite outcome of complications and 30-day mortality. These inconsistencies may be due to differences in study design or GDT protocols. The strength of the present study is the relative homogeneity of demographic characteristics, including study subjects, surgical procedures, and anesthetic techniques. In particular, the impact of GDT on hemodynamic management in such extensive surgeries has not been reported, to the best of our knowledge. Also, few studies have reported details and comparisons of MAP, HR, and urine output [12].

We observed trends toward higher MAP and HR in the GDT group and significant effects of GDT on these differences. The effect of GDT on HR can be reasonably explained based on the increased use of an inotrope. The effect of GDT on MAP indicated that GDT effectively maintained hemodynamic stability. The GDT protocol involved the use of an inotrope and vasoconstrictors to achieve cardiac output and MAP, respectively. This implies the presence of a non-trivial number of cases in the control group with low cardiac output and hypotension which were missed or untreated, and that such cases in the GDT group were more often treated using catecholamines, although clinical benefits of catecholamines have not been clearly demonstrated in the context of GDT.

The second important finding of the present study is a rank correlation between the amount of fluid administered and urine output in the GDT group, and a lack of such correlation in the control group. Few studies have reported such a correlating effect of GDT. This finding means that many patients in the control group received a large amount of fluid without increasing urine output. Urine output is traditionally regarded as an index of organ perfusion, and it is reasonable to hypothesize that these poorly responding patients are at higher risk for fluid excess. Conversely, in the GDT group, patients who received a larger amount of fluid tended to have a larger urine output. This correlating effect of GDT, combined with increased urine output, can be explained by the hypothesis that monitoring of a reliable predictor of fluid responsiveness and an estimation of cardiac output allows optimal use of fluids and inotropes, whereas inotropes maximize the hemodynamic effect of administered fluid. A previous meta-analysis of controlled trials of perioperative GDT focusing on renal outcomes found that GDT was the most beneficial in decreasing postoperative acute kidney injury when it was associated with the combination of an equivalent (not larger) volume of fluid and the use of inotropes [13] and explained this effect by the same reasoning.

The reduction of fluid balance with maintenance of hemodynamic stability and the correlation between the amount of fluid administered and urine output appear to explain the beneficial effect of GDT on the outcomes of surgical patients. A previous comparative study observed delayed recovery of gastrointestinal function in patients who received larger volumes of fluid after colon surgery [14]. Another study observed increased rates of cardiopulmonary and wound-healing complications after colon surgery in patients who received larger volumes of fluid [15]. The hypothesis that fluid restriction improves postoperative outcomes has been tested by many investigators, but the results are conflicting. Clearly, fluid restriction increases the risk of hypoperfusion. Nowadays, avoidance of fluid excess, rather than absolute restriction, is considered an important key to improve postoperative outcomes [16]. What constitutes “avoidance of fluid excess” is difficult to define, but background administration of relatively small volumes of fluids, combined with fluid loading triggered by signs of inadequate cardiac output and expected fluid responsiveness, is generally recommended [16, 17]. This is also a basis for recommendation of GDT in major surgery.

The present study has two major limitations. First, this is a single-center study with comparisons made to a historical control. Methods of hemodynamic and other perioperative management are highly variable between centers and between procedures, so we should be cautious before simply generalizing these results. Second, we did not assess patient outcomes such as postoperative complications or length of hospital stay, which would have improved the clinical relevance of our study. The present study was under-powered to draw conclusions regarding postoperative complications. We believe that studies with larger sample sizes and prospective design, including the collection of postoperative complication data, will address this limitation.

In conclusion, GDT in intraoperative management of PD increased urine output and reduced fluid balance while maintaining hemodynamic stability and resulted in correlation of urine output with the amount of fluid administered. These physiologic effects may be responsible for the beneficial effects of GDT on postoperative outcomes. Further characterization of such effects would help us improve intraoperative hemodynamic management.

## Abbreviations

ANOVA: Analysis of variance
ASA PS: American Society of Anesthesiologists Physical Status
BMI: Body mass index
CI: Confidence interval
eGFR: Estimated glomerular filtration rate
ERAS: Enhanced recovery after surgery
GDT: Goal-directed therapy
HR: Heart rate
MAP: Mean arterial pressure
PD: Pancreaticoduodenectomy
